# Deleterious Effect of Advanced CKD on Glyoxalase System Activity not Limited to Diabetes Aetiology

**DOI:** 10.3390/ijms19051517

**Published:** 2018-05-18

**Authors:** Lukáš Pácal, Katarína Chalásová, Anna Pleskačová, Jitka Řehořová, Josef Tomandl, Kateřina Kaňková

**Affiliations:** 1 Department of Pathophysiology, Faculty of Medicine, Masaryk University Brno, Kamenice 5, 625 00 Brno, Czech Republic; katarina.kuricova@gmail.com (K.C.); pleskacova@med.muni.cz (A.P.); kankov@med.muni.cz (K.K.); 2 Department of Biochemistry, Faculty of Medicine, Masaryk University Brno, Kamenice 5, 625 00 Brno, Czech Republic; tomandl@med.muni.cz; 3 Department of Gastroenterology, University Hospital Brno, Jihlavská 20, 625 00 Brno, Czech Republic; rehorova.jitka@fnbrno.cz; 4 Regional Centre for Applied Molecular Oncology, Masaryk Memorial Cancer Institute, Zluty kopec 7, 656 53 Brno, Czech Republic

**Keywords:** glyoxalase, diabetes, chronic kidney disease, diabetic nephropathy

## Abstract

Methylglyoxal production is increased in diabetes. Methylglyoxal is efficiently detoxified by enzyme glyoxalase 1 (GLO1). The aim was to study the effect of diabetic and CKD milieu on (a) *GLO1* gene expression in peripheral blood mononuclear cells; (b) GLO1 protein levels in whole blood; and (c) GLO1 activity in RBCs in vivo in diabetic vs. non-diabetic subjects with normal or slightly reduced vs. considerably reduced renal function (CKD1-2 vs. CKD3-4). A total of 83 subjects were included in the study. Gene expression was measured using real-time PCR, and protein levels were quantified using Western blotting. Erythrocyte GLO1 activity was measured spectrophotometrically. *GLO1* gene expression was significantly higher in subjects with CKD1-2 compared to CKD3-4. GLO1 protein level was lower in diabetics than in non-diabetics. GLO1 activity in RBCs differed between the four groups being significantly higher in diabetics with CKD1-2 vs. healthy subjects and vs. nondiabeticsfig with CKD3-4. GLO1 activity was significantly higher in diabetics compared to nondiabetics. In conclusion, both diabetes and CKD affects the glyoxalase system. It appears that CKD in advanced stages has prevailing and suppressive effects compared to hyperglycaemia. CKD decreases *GLO1* gene expression and protein levels (together with diabetes) without concomitant changes of GLO1 activity.

## 1. Introduction

Methylglyoxal (MGO) is a dicarbonyl aldehyde mainly formed as a by-product of glycolysis [1]. MGO production can be further stimulated by hyperglycaemia [2] in which an imbalance between MGO generation and elimination causes dicarbonyl stress with subsequent overproduction of certain types of advanced glycation end products (AGEs) [3]. MGO is efficiently metabolized by the glyoxalase system with the key limiting enzyme glyoxalase 1 (GLO1). The modulation of GLO1 activity is dependent on both regulation of gene expression and post-translational modifications. An adequate balance between MGO levels and GLO1 activity is necessary to ensure detoxification of MGO from different sources and cell survival [4]. Increased MGO formation or decreased GLO1 activity leads to the accumulation of AGE-modified proteins in the body which is the hallmark of many age-related diseases, such as obesity, cardiovascular and renal disease, or diabetes and its vascular complications [5,6,7,8,9]. Specifically, dicarbonyl stress significantly accelerates kidney aging, as well as kidney disease, and GLO1 has an important renoprotective effect [8]. Kumagai et al. showed that renal activity of GLO1 is significantly decreased by renal ischaemia-reperfusion injury in the rat model [10]. Giacco et al. generated non-diabetic mice with knocked-down GLO1 expression and found increased MGO concentration with subsequent alterations in kidney morphology similar to that caused by diabetes [7]. Furthermore, diabetic mice with GLO1 overexpression were completely protected from diabetes-induced oxidative stress and kidney pathology despite having hyperglycaemia [7]. Overexpression of GLO1 also reduces hyperglycaemia-induced oxidative stress in diabetic rats [11] and in cultured mouse renal mesangial cells [12]. A large body of literature supports the concept that MGO/GLO1 balance is crucial to maintain MGO levels below the toxic threshold.

Diabetic kidney disease (DKD) is a common complication of both type 1 and type 2 diabetes mellitus (T2DM) associated with significant morbidity and mortality and represents the most common cause of chronic kidney disease (CKD) nowadays. A number of mechanisms involved in DKD pathogenesis has been described, however, our knowledge is still insufficient to meaningfully improve renoprotective therapy and to stratify patients based on the risk of progressive CKD. The current treatment options target traditional risk factors and pathogenic mechanisms, including glycaemic control, hypertension, smoking, obesity, and inflammation [13,14].

We have previously studied another putative protective pathway in diabetes—a pentose phosphate pathway—capable of metabolizing proximal glycolytic intermediates with a potential to limit MGO to some extent production and, thus, alleviate intracellular damage due to hyperglycaemia. Although we have shown increased transketolase activity and intracellular thiamine diphosphate levels in CKD patients (either with or without diabetes) the expression of thiamine transporters was either decreased or unchanged. Therefore, a lack of adaptive increase of thiamine transmembrane transport allowing a further protective increase of transketolase activity might contribute to compromised protective mechanisms in diabetes and CKD and to the development of glycotoxic injury. Based on our previous results and recent evidence we hypothesize that diabetes in parallel with DKD might affect activation of the glyoxalase system in a similar manner, thereby causing sequential failure of protective pathways. Precisely, we intended to assess, in vivo, to what extent are the level and activity of GLO1 changed by diabetes and whether these changes are specific to diabetes or kidney dysfunction. Specific aims of the current study were to study the effect of diabetic and CKD milieu on a) *GLO1* gene expression; b) GLO1 protein level; and c) GLO1 activity in vivo in diabetic vs. non-diabetic subjects with corresponding CKD stages.

## 2. Results

Gene expression in PBMCs, protein level and activity of GLO1 in RBCs was compared between the 4 groups of subjects—diabetics and nondiabetics with or without CKD. As non-diabetic/CKD3-4 subjects were younger than remaining groups we first assessed correlations between age and all parameters studied and found none (all *p* > 0.05, Spearman correlation coefficient).

**Figure 1:**
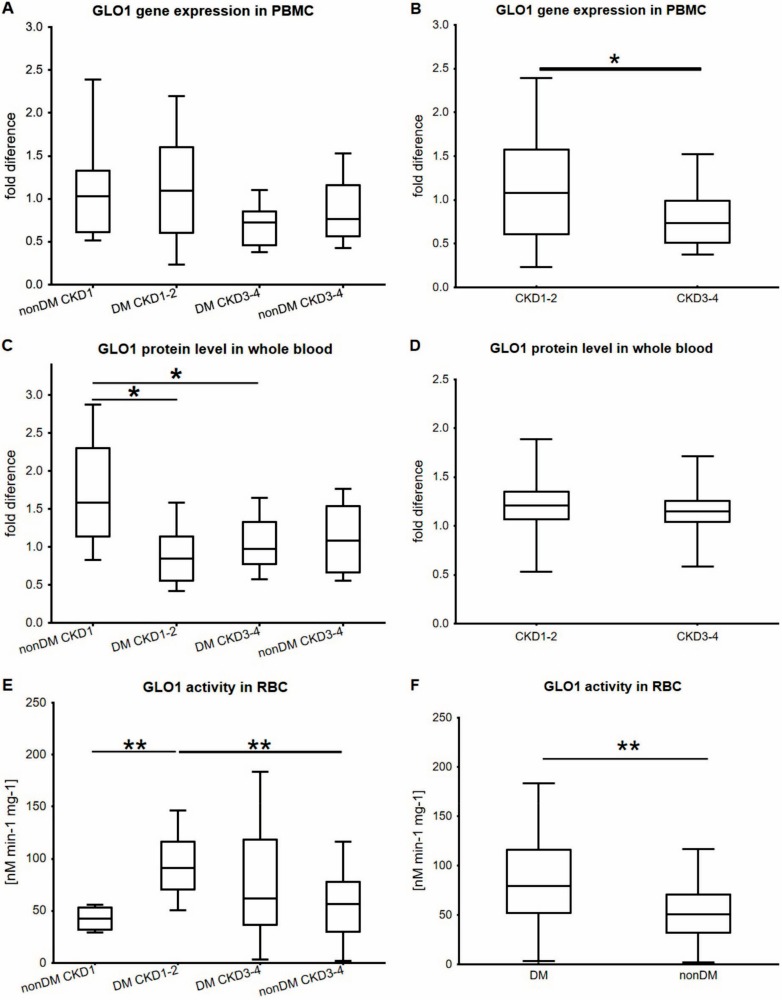
Fold differences in *GLO1* gene expression (**A**), protein level (**C**) and activity (**E**) between four groups of patients. Comparison of *GLO1* gene expression (**B**), protein level (**D**) and activity (**F**) between groups pooled based on renal status (**B**,**D**) and between subjects with and without diabetes (**F**). Box and Whisker plots were constructed as medians, minimum, and maximum values and interquartile ranges. Symbols * over the bar refer to significant differences between the experimental conditions (* *p* < 0.05, ** *p* < 0.01, Mann-Whitney test). DM—diabetes mellitus; CKD—chronic kidney disease; GLO1—glyoxalase; PBMC—peripheral blood mononuclear cell; RBC—red blood cell.

**Figure 2:**
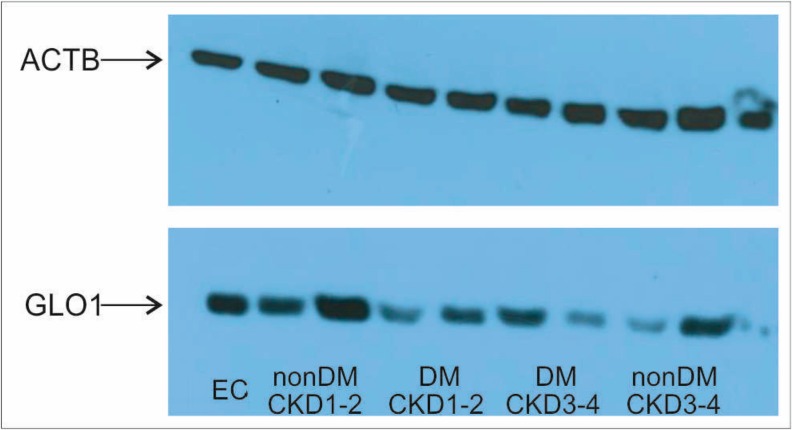
Representative Western blot of GLO1 protein. Figure shows from left endogenous control (EC) and two random patients from each group of subjects. DM—diabetes mellitus; CKD—chronic kidney disease; GLO1—glyoxalase; ACTB—β-actin.

Results for *GLO1* gene expression and protein levels are shown in Figure 1 and representative Western blot of GLO1 protein is shown in Figure 2. Despite non-significant differences in gene expression in PBMCs between the four groups, after pooling the groups based on the renal status we ascertained significant differences between subjects with CKD1-2 vs. those with CKD3-4 (expression being 1.5 times lower in CKD3-4; *p* = 0.033) (Figure 1A). Interestingly, when groups were pooled according to the presence of diabetes, *GLO1* gene expression did not differ between those with and without diabetes (data not shown). On the other hand, the protein level of GLO1 significantly decreased in diabetic patients compared to healthy subjects with intact kidney function (1.9 times; *p* = 0.011) and remained decreased in both diabetic and non-diabetic patients with CKD3-4 (1.6 times; *p* = 0.033 and 1.5 times; *p* = NS, respectively) (Figure 1B). Pooling groups based on renal status and presence of diabetes and subsequent comparison did not reveal any significant difference (data not shown). GLO1 activity in RBCs differed between the four groups being significantly higher in diabetics with CKD1-2 vs. healthy subjects (*p* = 0.0037) and vs. nondiabetics with CKD3-4 (*p* = 0.0033) (Figure 1C). GLO1 activity was significantly higher in diabetics vs. non-diabetics (*p* = 0.0077, Figure 1D), but did not differ between subjects with CKD1-2 and those with CKD3-4 (data not shown).

## 3. Discussion

Etiopathogenesis of microvascular diabetic complications is rather complex with several intracellular pathways activated by hyperglycaemia (and other metabolic alterations accompanying diabetes) in susceptible cell types established as pathogenic players. Among them, significant effort has been devoted to the study of dicarbonyl stress in diabetes and its vascular complications. Most studies focused on the role of AGEs, including kinetics of their precursor–MGO formation and on their interaction with receptors for advanced glycation end products. Less attention has, until recently, been dedicated to studies of potentially protective pathway opposing effects of harmful processes, such as dicarbonyl stress—namely, the glyoxalase system and enzyme GLO1, specifically. The direct pathogenic role of MGO/glyoxalase system in the development of diabetic nephropathy is strongly supported by animal experiments. Overexpression of GLO1 in diabetic rats reduced the production of AGEs, endothelial dysfunction, and also expression of early markers of kidney damage [15]. Interestingly, knockdown of GLO1 in nondiabetic mice induces kidney pathology very similar to diabetic nephropathy [7]. Furthermore, increasing plasma MGO in nondiabetic mice to the levels seen in mice with diabetes increased inflammation and vascular damage [16]. Given the above mentioned show that affected cells are able to mitigate the extent of hyperglycaemic/dicarbonyl damage by activation of protective pathways, and we can assume that damage develops as a result of the imbalance between damaging and protective pathways (likely in inter-individually variable manner). In our previous study we focused on this aspect by studying one of the proximal protective pathways—a pentose phosphate pathway (PPP)—and found the evidence of the lack of adaptive increase of thiamine (a crucial cofactor of key PPP enzyme transketolase) transport into the cells [17] irrespective of rising plasma thiamine levels paralleling the degree of kidney function in CKD [18].

In the present study, we were interested in whether gene expression, protein level, and activity of GLO1, a dominant component of another protective pathway—a glyoxalase system—might exhibit a similar pattern, i.e., a change as a response to two major metabolic derangements present in diabetes/CKD, which are hyperglycaemia and uraemia. Our major findings can be summarized as follows: We found that *GLO1* gene expression does not differ between the groups. However, comparison of subjects with CKD1-2 vs. those with CKD3-4 irrespective of diabetes showed significantly decreased *GLO1* expression in the latter group possibly as a result of uremic toxins accumulation. The effect of hyperglycaemia itself is less apparent as subjects with and without diabetes did not differ in *GLO1* gene expression. We also found that GLO1 protein levels were highest in healthy subjects compared to the remaining groups in which the presence of neither diabetes nor CKD created significant differences in GLO1 protein levels in pair-wise comparisons. Furthermore, comparing of pooled groups (either diabetic vs. nondiabetic or subjects with vs. without CKD) did not reveal significant differences. Finally, GLO1 activity was significantly increased by diabetes (of CKD1-2 stage) compared to nondiabetic controls with the same kidney function, but similar adaptive response was absent in both DM and non-DM CKD3-4 groups. Nevertheless, this finding requires further investigation since absolute quantification of GLO1 protein was not performed and activity was calculated using total protein content. Therefore, study performing parallel absolute quantification of GLO1 protein and activity, and ideally of MGO concentration in respective CKD stages, is definitely warranted. Taken together, our findings suggest that advanced CKD might preclude adaptive response of GLO1 and this might be a very substantial mechanism perpetuating development of organ complications in diabetes. To what extent are those findings mediated by presumably increased dicarbonyl stress has to be subsequently determined since MGO derivatives were not directly quantified in this study. Data concerning GLO1 in CKD/DKD are relatively scarce. To our knowledge, only one rather old study so far measured GLO1 activity and protein levels in subjects with compromised kidney function [19]. Both parameters did not differ between patients on haemodialysis and healthy control subjects, however sample size was rather small.

Our study has obvious limitation. The lack of internal GLO1 standard allowing absolute GLO1 protein quantification was already mentioned above. Furthermore, we were not able to measure plasma MGO or MGO-derived AGEs due to limited volume of biological samples. Nevertheless, evidence on abundancy of various AGE precursors and MGO levels in patients with defined CKD stages is available from literature. For example study measuring three α-oxoaldehydes [20] in subjects with CKD1-2, CKD3-5, CKD5D (each group comprised of patients with and without diabetes), and control subjects found a gradual increase of MGO and glyoxal, but not 3-deoxyglucosone with decreasing kidney function. Another study reported a significant negative correlation between MGO and creatinine clearance in patients with diabetes [21]. Finally, the role of AGEs in the pathogenesis of CKD has been documented by many original studies and comprehensively reviewed recently [22]. The authors summarised data supporting compelling evidence for the pathogenic role of dicarbonyl stress resulting from renal GLO1 down-regulation in CKD. Therefore, despite the lack of direct evidence for increased AGE levels in our study, we can reasonably suppose that attenuation of GLO1 in CKD stages 3-4 in our study is most likely related to increased dicarbonyl stress.

In conclusion, both diabetes and CKD affects glyoxalase system, however, in a somewhat conflicting manner. It appears that CKD in advanced stages has prevailing and suppressive effects compared to hyperglycaemia. CKD decreases GLO1 gene expression, together with diabetes and protein levels and—contrary to stimulatory effect of diabetes per se—is not associated with significant changes in GLO1 activity.

## 4. Materials and Methods

### 4.1. Human Study—Subjects, Study Design, and Criteria

The case-control study comprised a total of 83 subjects (48 men/35 women) classified by the presence or absence of T2DM and CKD stage as a secondary phenotype into four mutually comparable groups: (a) T2DM patients with normal or slightly decreased kidney function (CKD stage 1–2, *n* = 24), (b) T2DM patients with considerably reduced kidney function (CKD stage 3–4, *n* = 35), (c) non-diabetic patients with CKD stage 3–4 (*n* = 13), and (d) non-diabetic healthy subjects with normal kidney function (*n* = 11). Clinical characteristics of the study participants are shown in Table 1. Patients in groups (a) to (c) were enrolled from the clientele of the Nephrology and/or Diabetology units of Brno University hospital in 2014–2015. Inclusion criteria for diabetic patients were a T2DM duration of at least 10 years and GFR ≥60 mL/min/1.73 m^2^ for CKD1-2 group or GFR 59-15 mL/min/1.73 m^2^ for the CKD3-4 group. The latter GFR cut-offs were used for the nondiabetic CKD3-4 group as well (non-diabetic aetiology comprising IgA nephropathy, polycystic kidney disease, vasculitis, Wegener’s granulomatosis, and membranoproliferative glomerulonephritis in this particular group). Common comorbidities in patients with CKD3-4 were hypertension, coronary artery disease, and obesity. Healthy subjects were volunteers from the staff of the Faculty of Medicine, Masaryk University who had a history or clinical signs of neither diabetes nor renal disease and had eGFR (using CKD-EPI formula) ≥90 mL/min/1.73 m^2^. GFR was measured by creatinine clearance from 24-h urine collection in subjects of groups (a) to (c) and from a single measurement of plasma creatinine (obtained by cubital venepuncture see below) in group (d). The study was performed in accordance with the principles of the Declaration of Helsinki and was approved by the Ethical Committee of Faculty of Medicine, Masaryk University Brno (approval number 22/2010, date of approval 16.9.2010). Informed consent was obtained from all subjects prior to their inclusion in the study.

### 4.2. Blood Samples—Pre-Analytical Processing

A sample of peripheral EDTA-blood was taken from each participant from a single venepuncture, split into two tubes (2 × 7.5 mL) and immediately transported into the lab facilities where (without any delay) blood samples were processed further: (1) one of the 7.5 mL blood aliquots was used for separation of PBMC using Histopaque-1077 (Sigma-Aldrich, St. Louis, MO, USA) and subsequent gene expression analysis. Total RNA was extracted from PBMC using an RNeasy Kit (Qiagen, Hilden, Germany) according to the manufacturer’s instructions. To assess RNA purity the ratio of absorbance at 260 and 280 nm was used. One microgram (1 μg) of total RNA was reverse transcribed using a High-Capacity cDNA Reverse Transcription Kit (Applied Biosystems, Foster City, CA, USA) and stored at −20 °C until gene expression analysis. In parallel (2) another 7.5 mL blood aliquot was split further to obtain 2 × 500 μL of the whole blood (for protein quantification) and the remaining volume was centrifuged (1000× *g*, 10 min, 4 °C) to obtain RBCs (for GLO1 activity measurements). Whole blood samples and packed RBCs (lysed in distilled water 1:4 and aliquoted) were stored at −70 °C until further analysis.

### 4.3. Glyoxalase 1 (GLO1) Activity Measurement

GLO1 activity was measured as previously described [23] with modifications. Briefly, hemithioacetal was formed in situ by pre-incubating 2 mM reduced glutathione and 2 mM methylglyoxal for 10 min in 50 mM sodium phosphate buffer (pH 6.6) at 37 °C. Enzymatic activity was measured spectrophotometrically by following the increase in absorbance at 235 nm (Δε_235_ = 1.07 mM^−1^ cm^−1^) on a Cytation3 (BioTek, Winooski, VT, USA) in 96-well UV-microplates (Corning, Corning, NY, USA) at 37 °C for 10 minutes (formation of S-D-lactoylglutathione). The GLO1 activity was expressed as nanomoles of substrate per litre per minute per milligram of total protein. Total protein concentration in diluted RBCs lysates was determined by commercial protein assay (Bio-Rad, Hercules, CA, USA) according to the manufacturer’s instruction.

### 4.4. Gene Expression Analysis

Real-time PCR was carried out on an ABI PRISM 7000 system (Applied Biosystems, Foster City, CA, USA) using TaqMan™ Gene Expression Master Mix (Applied Biosystems, Foster City, CA, USA) and predesigned hydrolysis probes (TaqMan Assays Hs00198702_m1 for GLO1, Applied Biosystems, Foster City, CA, USA) to determine the level of gene expression. The amplification programme consisted of a 95 °C (10 min) hot start followed by 40 cycles of 95 °C (15 s) and 60 °C (60 s). Each reaction contained 50 ng of cDNA, 1 μL of predesigned hydrolysis probe, 12.5 μL of master mix, and water in the total volume of 25 μL. The efficiency of PCR ranged from 90 to 100%. Data were normalized to a reference gene, *β*-*actin* (ACTB, TaqMan assay Hs99999903_m1, Applied Biosystems, Foster City, CA, USA). Relative gene expression was analysed using the comparative Ct method (2^−ΔΔCt^ method).

### 4.5. Protein Isolation and Western Blotting

For protein isolation, whole blood aliquots were lysed with water and haemoglobin was removed using HemogloBind™ (Biotech Support Group, Monmouth Junction, NJ, USA) according to the manufacturer’s instructions with the addition of a protease inhibitors cocktail (Protease Inhibitor cOmplete Mini, Roche, Basel, Switzerland). Protein concentrations were determined using a BCA protein assay (ThermoFisher Scientific, Waltham, MA, USA). Protein samples were then diluted to a final concentration 2 μg/μL with 2× Laemmli sample buffer (Bio-Rad, Hercules, CA, USA) and stored at −20 °C until analysis. Twenty micrograms (20 μg) of protein lysates were separated in 1.5 mm thick 12% SDS-PAGE gels. Proteins were transferred onto Immobilon-P transfer membrane with 0.45 μm pores (Merck Millipore, Burlington, MA, USA) using wet transfer (4 °C, 90 min, 100 V). Membranes were blocked in blocking buffer (5% dry milk in PBS with 0.01% Tween) for one hour and incubated overnight at 4 °C with respective primary antibodies diluted in PBS with 5% dry milk and 0.01% Tween. The following antibodies and dilutions were used: anti-GLO1 (Sigma-Aldrich, St. Louis, MO, USA, SAB4200193, 1:4000) and anti-β-actin as a reference protein (Sigma-Aldrich, St. Louis, MO, USA, A5441, 1:5000). After incubation membranes were washed four times for 10 min in PBS buffer and then incubated for 60 min at room temperature with respective secondary antibody: anti-rat (Dako, Santa Clara, CA, USA, P0399, 1:4000) or anti-mouse (Dako, Santa Clara, CA, USA, P0260, 1:5000). An endogenous control sample was loaded in the first line of each gel for the quantification of densitometric data [24]. Protein samples isolated from whole blood of a single donor served as an endogenous control (EC). Results were analysed using ImageJ software (National Institutes of Health, USA, 1.48v). Relative, normalized protein levels were calculated by the NDL/FD method [24]. The density of target protein (TP) in each sample was multiplied by the ratio between the density of the loading control (ACTB) in endogenous control (EC) and this sample (S). This will give the normalized density to the loading control (NDL = TP(s) × ACTB(EC)/ACTB(s)). By dividing NDL from each sample by the NDL from the endogenous control sample we calculated the fold difference (FD = NDL(s)/NDL(EC)).

### 4.6. Statistical Analysis

Results are expressed as the average of the 2^−ΔΔCt^ value ± standard deviation (SD) in gene expression analysis and the average of the fold difference ± SD in protein analysis. Statistical analysis was performed using Statistica 12 (StatSoft, Tulsa, OK, USA). Differences between groups were evaluated using Kruskal-Wallis or Mann-Whitney tests. *p* < 0.05 was considered statistically significant.

## Figures and Tables

**Table 1:** Clinical characteristics of the subjects.

Parameter	Non-DM CKD1	T2DM CKD1-2	T2DM CKD3-4	Non-DM CKD3-4	*p*
	(*n* = 11)	(*n* = 24)	(*n* = 35)	(*n* = 13)	
Age (years)	65.0 (52.0–77.0)	64.5 (60.5–67.0)	67.5 (64.0–75.0)	53.0 (31.0–67.0)	<0.01
HbA1c (mmol/mol)	---	57.0 (49.5–68.0)	57.0 (50.0–73.5)	---	NS
DM duration (years)	---	13.5 (11.5–16.5)	17.0 (10.0–25.0)	---	NS
Creatinine (µmol/L)	70.8 (49.0–86.0)	85.5 (75.5–103.0)	199.5 (160.0–250.0)	212.0 (140.0–371.0)	<0.01
GFR (mL/s)		1.82 (1.16–1.87)	0.55 (0.38–0.78)	0.51 (0.34–0.79)	<0.01
Glucose (mmol/L)	5.15 (4.5–5.25)	6.95 (5.8–8.5)	8.3 (6.0–11.6)	5.0 (4.3–5.5)	<0.01
CKD-EPI (mL/s/1.73 m^2^)	---	1.2 (0.99–1.45)	0.46 (0.3–0.57)	0.36 (0.23–0.63)	<0.01
GLO1 activity (nmol/min/mg)	42.3 (31.85–53.2)	90.9 (70.7–116.1)	62.0 (36.4–118.3)	56.7 (30.1–77.75)	<0.01

Data are presented as median and lower and upper quartiles. Data were analysed using Kruskal-Wallis test. HbA1c—glycated haemoglobin; DM—diabetes mellitus; GLO1—glyoxalase 1; GFR—glomerular filtration rate; Hb—haemoglobin; CKD—chronic kidney disease; NS—not significant.

## Abbreviations

AGE	Advanced Glycation End Product
CKD	Chronic Kidney Disease
DM	Diabetes Mellitus
DKD	Diabetic Kidney Disease
GFR	Glomerular Filtration Rate
GLO1	Glyoxalase 1
HbA1c	Glycated Haemoglobin
MGO	Methylglyoxal
PBMC	Peripheral Blood Mononuclear Cell
PPP	Pentose Phosphate Pathway
RBC	Red Blood Cell
T2DM	Type 2 Diabetes Mellitus
